# Shared Etiology in Autism Spectrum Disorder and Epilepsy with Functional Disability

**DOI:** 10.1155/2022/5893519

**Published:** 2022-04-27

**Authors:** Aqeela Zahra, YunFu Wang, Qun Wang, Jianping Wu

**Affiliations:** ^1^School of Chemistry, Chemical Engineering, And Life Sciences, Wuhan University of Technology, Wuhan 430070, China; ^2^Taihe Hospital, Hubei University of Medicine, 430070, China; ^3^Beijing Tiantan Hospital, Capital Medical University, Beijing 100070, China; ^4^Advanced Innovation Center for Human Brain Protection, Capital Medical University, Beijing 100070, China; ^5^National Clinical Research Center for Neurological Disease, Beijing 100070, China

## Abstract

Autism spectrum disorders and epilepsies are heterogeneous human disorders that have miscellaneous etiologies and pathophysiology. There is considerable risk of frequent epilepsy in autism that facilitates amplified morbidity and mortality. Several biological pathways appear to be involved in disease progression, including gene transcription regulation, cellular growth, synaptic channel function, and maintenance of synaptic structure. Here, abnormalities in excitatory/inhibitory (E/I) balance ratio are reviewed along with part of an epileptiform activity that may drive both overconnectivity and genetic disorders where autism spectrum disorders and epilepsy frequently co-occur. The most current ideas concerning common etiological and molecular mechanisms for co-occurrence of both autism spectrum disorders and epilepsy are discussed along with the powerful pharmacological therapies that protect the cognition and behavior of patients. Better understanding is necessary to identify a biological mechanism that might lead to possible treatments for these neurological disorders.

## 1. Introduction

Autism spectrum disorders (ASD) and epilepsy are two conditions with distinct pathophysiology [1]. ASD is a complex developmental condition involving persistent challenges with social communication, restricted interests, and repetitive behavior [2]. Autistic patients have higher rates of co-occurring learning disability, language deficit, and seizure than the general population [3]. Severe epileptic encephalopathy abnormalities in early childhood appear to be associated with ASD [4]. Such individuals have many uncontrollable seizures due to structural aberration that may lead to ASD [5]. Additionally, epileptic symptoms similar to those of ASD patients may comprise neurodevelopment impairments, language disorders, cognitive disabilities, excitatory and inhibitory (E/I) ratio imbalances, altered circadian rhythms, and gastrointestinal pain [6].

The occurrence of seizures in children may lead to alteration in various brain regions that adversely affect autism, and related maladaptive behaviors [7] and seizures have strong and variable effects on ASD patients. It is important to determine the outcomes of seizures and understand how they influence patients. Research into such topics can aid in understanding the coexistence of ASD and epilepsy, as well as in the diagnosis, measurement, and treatment of patients. For instance, many researchers identify those specific symptoms or behaviors commonly exhibited by individuals with epilepsy, and ASD may indicate specific brain regions damaged by seizures.

Genetic generalized epilepsies (GGE) comprise roughly 20% of epilepsy diagnoses and include absence seizures (AS), myoclonic seizures (MS), and generalized tonic–clonic seizures (GTCS) [8]. Quantitative MRI investigations have found structural abnormalities in cortical and subcortical areas of the brain when comparing patients with healthy controls [9]. The majority of investigations have found a reduction in the volume of the thalamus in GGE [10]. Additionally, some studies show an increase in volume [11] or no significant difference in volume of the thalamus in GGE patients [12].

Besides the seizure prognosis, cognitive and behavioral comorbidities are common and can have negative psychosocial outcomes [13]. There is a correlation between more severe symptoms and longer-lasting epilepsy and antiepileptic medication treatment in children with AS, which is also known as childhood absence epilepsy (CAE). Up to 37% of children with CAE are diagnosed with attention deficit–hyperactivity disorder (ADHD), with greater risk of longer epileptic duration and higher seizure frequency [14]. MS has been documented in Juvenile myoclonic epilepsy (JME), has problems with word fluency and interruption, poor planning, and task shifting capabilities, and deficits in memory tests [15]. Anxiety and affective disorders are the most common within GEE, followed by personality disorders, and subsequently schizophrenia and other psychotic diseases. Multiple factors, such as the underlying channelopathy, brain structural changes, and the impact of repetitive epileptiform discharges, are major contributor to these comorbidities.

**Figure 1:**
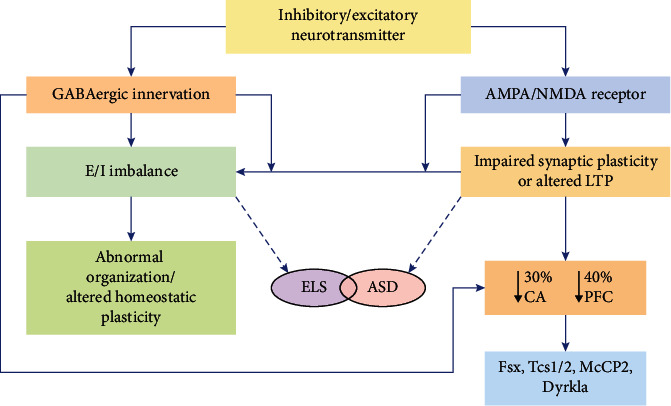
Genetic pathologies linked with epilepsy and ASD. Synaptic plasticity can be altered by epilepsy or seizures in an early phase of infantile development leading to ASD. Anomalies in neural plasticity can be a result of malfunctioning neurotrophins, signaling molecules, or receptors, resulting in a 30% reduction in dendritic spine density in hippocampus (Cornu Ammonis) CA3 neurons and a 40% reduction in prefrontal cortex (PFC) neurons. Several genes involved in autism and epilepsy pathogenesis have been linked to this biochemical pathway critical in brain development and function.

Since epilepsy and autism are common comorbidities, differential changes in IL-6 and IL-12p40 were found to be associated with electroencephalogram (EEG) findings and suggest that the downregulated expression of IL-6 in combination with the upregulated expression of IL-12p40 may be an element related to the risk of epilepsy comorbidity in children with autism [16]. Autistic behavior is mainly caused by a lack of connection between the frontal and posterior brain areas, which is predicted to have an influence on activities that require significant synchronization among frontal and posterior processing centers [17]. Imaging studies of individuals with ASD have found that numerous brain areas, including the frontal cortex, striatum, hippocampus, amygdala, and unusually tiny and densely packed neurons in the thalamus and cerebellum, are affected [18]. Evidence suggests that ASD and epilepsy may be implicated as a condition associated with synaptic plasticity as a result of an imbalanced E/I ratio in the developing brain as shown in Figure 1. In what manner does this outcome amplify any correlation between ASD and epilepsy? In the developing brain, this may occur due to genetic mutation causing an imbalanced excitability ratio and impaired synaptic plasticity [19]. Synaptic plasticity is altered due to changes in receptors and signaling channels, including receptor molecules and neurotrophins. Correspondingly, an early-life seizure has several genetically altered mutations in many molecules known to be related to ASD and epilepsy.

These processes are examined here by reviewing common features of ASDs and epilepsies, including gene transcription control, cell proliferation, and development and synaptic growth [20]. It is proposed here that if epilepsy and autism are not considered a single condition due to their various expressions, then none of the conditions should be considered individually. These studies would contend that both seizures and autism play a major role in the onset of neurocognitive problems [21], whereas the existing data on connectivity is predominantly from fMRI or structural approaches by diffusion imaging, and complementary methods have been increased [22]. EEG/MEG functional connectivity will show qualitatively distinct findings that are critical in understanding how autism affects brain wiring. Here, evidence on the relationship between epilepsy or EEG function and E/I balance in autism and coexisting genetic disorders is comprehensively reviewed.

## 2. ASD and Epilepsy: General View (Prevalence and Risk Factors)

The frequency of epilepsy associated with ASD is highly variable. Studies indicate that about 50% of those with ASD have epilepsy [23], and the Centers for Disease Control and Disease Prevention report that between 1 in 88 and 1 in 100 people are affected [24]. Numerous clinical trials demonstrate a maximum percentage since epileptic patients are often overrepresented in certain cases [25]. Although population analyses provide objective data and include the most reliable estimates of the true prevalence of epilepsy and autism, the reported rates remain inconsistent [26]. A meta-study of data obtained between 1963 and 2006 revealed that individuals with ASD and intellectual impairment (ID) shared a 21.5% prevalence of epilepsy; while comparably, epilepsy was prevalent in 8% of ASD lacking ID [27]. In children with ASD, a group correlated with a higher degree of cognitive dysfunction and brain epileptiform development [28] indicates coexistence with epilepsy in comparison to children with ASD alone. Thus, progressive infantile spasms signify an elevated risk of the presence of autism [29].

Additionally, an increased prevalence of interictal abnormalities in ASD was found in EEGs [30]. Numerous recent studies have reported that offspring with ASD exhibit interictal spikes comprised up to 60% of EEG recordings [31]. Often children with inconsistent EEGs have no epilepsy report [32]. Additionally, it has been reported that higher ID correlates with an increased risk of epilepsy, and that the relationship between autism and epilepsy is statistically explained by moderate to severe ID [33]. In short, it has been shown that neurodevelopmental disorders including epilepsy are often associated with autism, with an increased risk of disease and death in people with seizures [34].

According to the epileptic encephalopathy hypothesis, seizures and epileptic function can potentially coexist in the presence of neurological disorders. Epilepsy and its activity have a greater impact on cognition and behavior than underlying neurodevelopmental dysfunctions [35]. Additionally, mortality risk is greater in epileptic individuals with treatment resistant epilepsy and is associated with developmental deficits [36]. Numerous studies on the early development of epilepsy have been conducted due to the complex relationship involving seizures similar to convulsive activity, autism, and ID. Evidence has shown that 14% of children with early-onset epilepsy develop autism indicate that early seizures are more susceptible to the development of autism and intellectual impairments [37].

Nonetheless, the coexistence of early-onset epilepsy, autism, and ID remains unresolved as both epilepsy and autism are indistinguishable in terms of their inherent pathologies and the associated implications for cognitive and social skills. Currently, it is hypothesized that epilepsy and autism, as well as ID, often coexist. Additionally, it is suspected that children with early-life seizures may exhibit an elevated risk of developing autism, which often involves cognitive deficiency.

## 3. Neural Coordinating Theory

### 3.1. Epileptiform Activity in ASD

The association between ASD and epilepsy indicates the presence of an underlying encephalopathy manifested by a variety of neurologic disorders, including pathological epileptiform activity. Studies of EEG abnormalities and seizures in autistic children provided the first recorded evidence of autism's neurological etiology. Extensive research on 147 children with autism shows that 64% had irregular brain wave activity in EEGs. The repeated observation of epileptiform activity in individuals with ASD, often in the absence of clinical epilepsy, has raised the possibility that this activity is etiologic, rather than comorbid. Rett, Fragile X, Angleman, and Prader-Willi syndromes, as well as other pediatric epileptic encephalopathies, frequently have both epileptiform and ASD symptoms. It has been suggested that irregular EEG patterns contribute to the behavioral disruptions seen in ASD patients [38]. Interictal EEG abnormalities are often thought to contribute significantly to behavioral comorbidity. Data indicate that interictal abnormalities may impair visual, cognitive, and higher brain functions such as language abilities in both humans and animal models [39].

There is a paucity of data on the relationship between interictal epileptiform discharges and ASD. The majority of studies have suggested that there is a significant rate of comorbidity with respect to interictal discharge and autism [40]. One study noted that early epileptiform activity predisposes a patient to symptoms such as decreased plasticity and insufficient neural networks, which result in impaired cognitive, social, and stereotyped behaviors associated with autism [41]. Its findings indicated that the incidence of autism symptoms could be correlated with an increased risk of developing epileptiform abnormalities, however, whether therapy affects outcomes was uncertain.

The development of autism behavior was substantially linked to frontal anomalies in EEGs primarily characterized as bilateral and chronic hypsarrhythmia. The authors hypothesized that paroxysmal discharges in rapidly maturing cortical areas can contribute to the development of autistic characteristics [42]. Correspondingly, it has been shown that the existence of frontal paroxysm is substantially more indicative of future epilepsy progression than centrotemporal paroxysms [43].

### 3.2. E/I Ratio in Epilepsy and Autism

Abnormal or insufficient inhibition results in hyper excitability of the nervous system, a phenomenon that can lead to seizures. So far, this idea has been crucial in helping investigators to understand more about epigenetic changes, ictogenesis, and treatment planning. In fact, seizures may be explained in many epilepsy syndromes by either a loss of inhibition, as in GABAergic receptor or interneuron dysfunction, or an increase in excitatory pathways. According to the E-I balance theory, GABAergic agonists and sodium or calcium channel blockers are highly effective therapies for seizures [44]. The E-I balance notion is crucial since it provides a functional paradigm with clearly established implications, such as the suppression of seizures.

Investigation of neonatal seizures (NS) suggests that an altered E/I equilibrium leads to autism like behavior in rodents [45]. It has recently been discovered that initially seizures can modify the role of neurotransmitters and intrinsic neuronal properties that lead to cognitive impairment and learning disabilities [46].

There are several potential effects of epilepsy and epileptogenesis on synaptic plasticity in the developing brain. LTP and memory configuration are disrupted by the development of GABA-A receptors acting with benzodiazepines [47]. It is known that GABA-A receptor ɑ subunits are the major modulator in a crucial phase of spatial learning [48] and synaptic plasticity in the hippocampus [49]. When compared to adult rats showing decreased *α*-subunit expression following pilocarpine-induced seizures, the type I benzodiazepine receptor upregulation is either responsible for or linked with these functional alterations [50]. Following the preceding discussion, it is recommended that the excitation of GABA-A receptor subunits depends upon age and is affected by seizures, while increased inhibition can facilitate cognitive impairments by the amplified GABA-A receptor expression following NS [51].

Both excitatory synaptic density and activation occur following NS, mediated by AMPA and NMDA receptors. Dendrite spine density in CA3 neurons is reduced by approximately 30% following tetanus toxin induced seizures during the fetal period [52], and NMDA receptor subunit proteins NR1, NR2A, and NR2B exhibit a 30-40% reduction in the hippocampus [53]. Following prenatal lithium pilocarpine and hypoxia induced seizures, it was discovered that the AMPA receptor GluR2 subunit expression was decreased [54]. In the medial prefrontal cortex (mPFC), NS has been shown to strengthen short-term plasticity, and alteration in hippocampal-PFC synchrony is also correlated with modified short-term plasticity. This indicates that the mPFC exhibits long-term amplification of the E/I ratio along with NS [55]. Thus, rodent models exhibit irregular social tendencies [56] and behavioral deficiencies [57] similar to those seen in children with autism.

Similarly, the E/I imbalance ratio induces abnormal microcolumn organization, alteration in metabolic pathways [58], homeostatic plasticity or alterations of glial function, and many other findings characteristic of ASD syndromes. In syndromic families comprising SCN1A [59], GRIN2A mutations [60], PTEN [61], and others, numerous genetic conditions have been identified to amend the E/I balance.

Pleiotropy is frequently reported, with a single mutation causing a range of symptoms (from moderate to severe, with occasionally incomplete penetrance) [62]. Seizures are considered as an important factor for phenotype worsening in epilepsy, particularly in epileptic encephalopathies, in which epileptic activity is thought to strongly contribute to cognitive and behavioral impairments [63]. SCN1A mutations are significant examples of these issues: they develop Dravet syndrome (DRS), a severe form of epileptic encephalopathies, and genetic epilepsy with febrile seizures plus (GEFS+), a milder form of epileptic encephalopathies with a high degree of phenotypic heterogeneity (e.g., around 3% of GEFS+ individuals develop DRS) [64]. It has been suggested that the mutation-induced sodium channel SCN1A (NaV1.1) dysfunctions may be the direct source of behavioral and cognitive abnormalities in mice, supporting the concept that DRS is a pure channelopathy and a developmental encephalopathy (DE), instead of an epileptic encephalopathies [65]. Another study compared the effects of short seizures generated at the age of disease onset in SCN1A models with hyperthermia in SCN1A RH/+ mice and with the convulsant flurothyl in SCN1A RH/+ mice and WT littermates [66]. These findings indicate that short recurring hyperthermic seizures can trigger neuronal excitability remodeling in SCN1A RH/+ mice, altering their phenotype from mild/asymptomatic to severe DRS-like. Furthermore, hyperexcitability of excitatory neurons is detected solely in SCN1A mice models that have undergone seizures, corresponding with seizure-induced pathological alteration of specific age and neuron subtypes. These results do not support the concept of DRS as a pure channelopathy or DE, but they do suggest that seizures are an important contributor to the development of severe phenotypes in carriers of SCN1A variants, and that mutations/variants may increase the risk that seizures cause harmful effects on the brain.

In the NS model, mPFC and hippocampus were monitored using multisite local field potentials within and among brain sites associated with cognition in an effort to understand the effects of E/I imbalance in autism. Similarly, the NS mouse model exhibits anxiety-like behavior and impaired social tendencies as compared to control suggesting inadequate synaptic plasticity [45]. Brain connectivity and neural plasticity changes are proposed as mechanisms that lead to autistic like symptoms manifesting as increased coherence over a broad frequency range [67].

Recently, several studies have diverged from the current paradigm of shared pathogenicity among epilepsy, autism and ID [68]. The consistency of related genetic variants seen in ID, autism, and epilepsy may help to explain the mutual pathogenicity of these phenotypes. A recent trial found a physiological connection between long-range frontal circuits and genotypes of CNTNAP2, by using physiological neuroanatomy combined with gene expression [69]. The CNTNAP2 influences autism by altering frontal lobe connectivity. This model of using gene expression and physiological neuroanatomy can widen understanding of genetic influences on neural development and illuminate why different neurodevelopmental pathologies like ID, epilepsy, and autism share the same phenotypical characteristics.

## 4. ASD/Epilepsy Coexisting Genetic Syndromes

Synaptic plasticity is the mechanism by which synapses are reinforced by an event or experience. Numerous proteins are involved in synaptic plasticity in autism and epilepsy, and their genes are disrupted as a result of genetic mutations [70]. Recently, it was discovered that some common genes associated with autism, epilepsy, and ID share potential genetic mechanisms underlying epileptic seizures and cognitive impairments [71]. ASD and epilepsy have been linked to a range of disorders caused by genomic copy number variation or single gene mutations [72]. Numerous illustrations are given in Table 1 and discussed briefly in the following section.

### 4.1. Tuberous Sclerosis Complex (TSC) (Single Gene Mutation)

**Figure 2:**
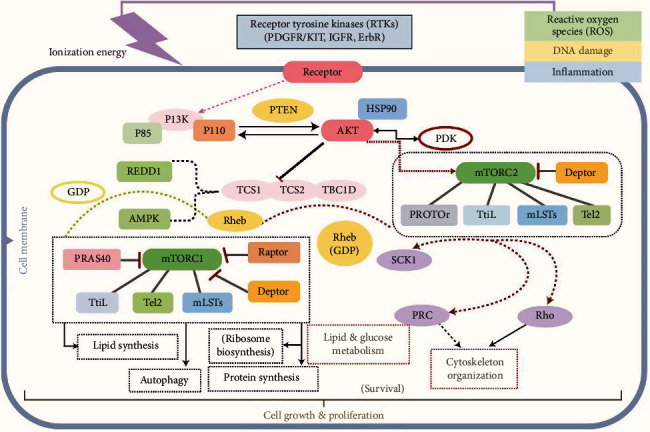
PI3K/AKT/mTOR pathway. Receptor tyrosine kinase activates the mTOR pathway. Hyperactivation of mTOR1 occurs due to TSC1/2 mutations. Downstream signaling pathway results in lipid and protein synthesis, ribosome biosynthesize, and autophagy. Similarly, mTOR2 downstream signals to lipid and glucose metabolism and cytoskeleton organization which accelerated cell division, proliferation, and irregular gene expression that exemplify TSC; RTKs: receptor tyrosine kinase; PDGFR: plate-let-derived growth factor receptor; KIT: protooncogene receptor; IGFR: insulin-like growth factor receptor; mTOR: mammalian target of rapamycin; PI3K: phosphatidylinositol 3-kinase; PTEN: phosphatase and tensin homologue deleted on chromosome 10; RHEB: Ras homologue a small GTP-binding protein enriched in the brain; TSC: tuberous sclerosis.

In neurodevelopmental disorders, TSC suggests a traditional approach for recognizing and initiating the relationship between epilepsy and ASD. Severe symptoms of TSC include neonatal spasms, repetitive seizures, cognitive impairments ranging from mild learning disabilities, and behavioral deficiencies to autistic spectrum disorder and behavioral deficits [100]. Mutation in hamartin or tuberin (programmed via TSC-1 and TSC-2 genes), which mutually reduces the signaling pathway of phosphatidylinositol 3-kinase (PI3) as well as cell growth, proliferation, and protein translation, is stimulated by mTOR, translational elements, and many second messenger kinases as shown in Figure 2. TSC-related lesions throughout the body are mainly due to dysregulation in mTORC1. Particular pathways of autism and epilepsy in TSC are yet unidentified. In mice, the E/I imbalance in TSC induces both autistic like characteristics and epilepsy [101]. Applied approaches and the overall prevalence of ASD in TSC lies between 26-45% [102].

**Figure 3:**
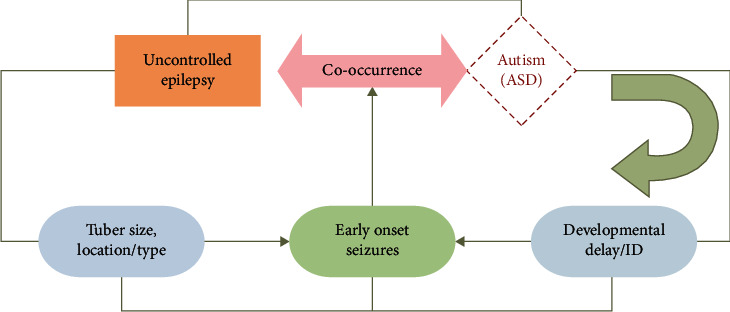
Significant characteristics in TSC. This demonstrates the correlation between phenotypic characteristics and predictive risk factors in patients with TSC.

Numerous characteristics like early-onset seizures, cyst like tubers, TSC2 mutation, brain lesion, the volume and size of the tubers, prominent lesion type, and severe cognitive impairment are related to ASD in TSC as shown in Figure 3. An individual with no ASD has fewer recurrent seizures as well as early age seizure onset, and their EEGs had a decreased ratio of interictal epileptiform properties in the left temporal lobe relative to ASD patients. It is reported (*n* = 29 Japan) that everolimus therapy radically decreases the rate of seizure frequency in TSC patients with the increase in developing possibilities of ASD. Similarly, another investigation evaluated that everolimus therapy in TSC patients showed improvements in ASD symptoms such as social skill, verbal communication, and recurring actions [103]. It has also been proven that early-onset seizures have a role in the development of ASD and TSC.

Another study conducted in the Netherlands on 32 children with TSC found that everolimus did not affect either cognitive or psychomotor functioning or autism characteristics when compared to a placebo. Because the median age of patients receiving placebo was 11.5 years and the median age of patients receiving everolimus was 12.2 years in that study, no solid conclusions were made [103]. There is a need for formal research to establish whether this is the cause of early-life seizures and brain development; nevertheless, early therapy with everolimus may be needed for improvement in characteristics such as social interaction, language, and repetitive behavior.

To identify primitive biomarkers for epilepsy therapy, organizations such as TACERN (Tuberous Sclerosis Complex Autism Center of Excellence Network) and (epileptogenesis in a genetic model of epilepsy–tuberous sclerosis complex) EPISTOP classified the predictive characteristics of epilepsy and autism throughout the TSC [104]. As a result, the increased risk of ASD associated with TSC can be mitigated but not eliminated. While both epilepsy and ASD are amenable to antiepileptic action, they share a similar pathophysiological mechanism that contributes to an infant's vulnerability to either disorder.

### 4.2. Fragile X Syndrome (FXS) (Single Gene Mutation)

FXS is another paradigm of a genetic condition with an elevated occurrence of ASD with epilepsy. This genetic syndrome is mainly a recurrent type of inherited cognitive disability that frequently occurs in autistic and epileptic individuals. FXS is primarily described by the existence of sufficiently long and thin dendritic spines and immature neural architecture [105]. FMRP, the mRNA binding proteins regulated by FMR1 genes, are abundant in brain connective tissue and modulate up to 4% of other RNAs, implying neural plasticity. Each mutation in the FMR1 gene contributes to the reduction of FMRP release [106]. FMRP also synchronizes the transport of mRNA in dendrites [107]. FMRP deficiency results in excessive and irregular mRNA translation, decreased synaptic function, and a deficiency in synaptic plasticity regulating proteins. FMRP is involved in the wiring of neuronal pathways, which is highly complex and depends on a sequence of events during neuronal growth such as axonal proliferation and neural configuration [108].

Interestingly, FMRP knockout mice display autistic-like characteristics [109] such as decreased synaptic activity, immature dendrite architecture, and impaired cognition, suggesting that FMRP plays an important function in modulating and stabilizing synaptic plasticity [109]. Furthermore, in FXS, glutamatergic neuron dysregulation disrupts the decreased function of GABA receptor subunits and the atypical activities of inhibitory GABAergic neurons. Multiple GABA remodeling enzymes seen in FXS have been altered to facilitate hyperexcitation and seizures.

### 4.3. Rett Syndrome (RTT) (Single Gene Mutation)

RTT is a postpartum neurodevelopmental condition that is more evident in females during childhood. The symptoms of RTT emerge early at the age of 6–18 months and comprise language disability, lack of social skills, and persistent motor disability. MeCP2 is a transcription factor involved in chromatin modulation and the genes responsible for RTT encode RNA splicing. In resting neurons, MeCP2 binding to methylated CpG dinucleotides regulates the gene expression through histone deacetylase corepressor complexes and chromosome modification proteins. It was discovered that MeCP2 acts as a transcriptional activator for a variety of genes [110].

In RTT, lack of MeCP2 results in altered gene manifestation by loss of activity that could disrupt neural plasticity [83]. The absence of MeCP2 has been shown to alter the expression of thousands of genes [111], but the precise mechanism accompanying MeCP2 deficiency in epilepsy and ASD is still unknown. In the developing brain, these changes trigger an aberration in synaptic plasticity, subsequent to an abnormal E/I ratio. Additionally, genetic mutations that contribute to seizures and epileptogenesis in early life impair synaptic plasticity and inevitably lead to ASD and ID [112].

### 4.4. Down Syndrome (DS) Copy Number Variation

DS is described by distinctive facial dysmorphisms, intellectual impairment, and congenital abnormalities. Epilepsy occurs in about 8–13% of individuals with DS [113]. Patients with DS have been documented to experience a variety of different seizure types. Children who have both DS and ASD can experience a general decline in brain function and an increased risk of seizures. It is suggested that 5–9% of individuals with DS exhibit ASD-like symptoms [114]. In offspring with DS, diagnosing ASD remains a concern due to comorbid intellectual impairment. Comparing 20 children with or without ASD with trisomy 21, it was discovered that those with ASD have slightly more impaired language skills, social ability, and cognition [115]. In mouse models, Dyrk1A has been shown to perform critical roles in cell cycle regulation and synaptic plasticity [116]. Furthermore, whole exome sequence study has detected Dyrk1A mutation in many ASD and microcephalic infants [116].

### 4.5. Phelan-McDermid Syndrome/SHANK3 Deletion

SHANK3 is a scaffolding protein located in the postsynaptic region that regulates the production of the metabotropic glutamate receptor 5 (mGluR5) [117]. Shank3 also plays a role in the regulation of AMPA receptors recycling and synaptic long-term potentiation [118]. Mutation of the SHANK3 gene at 22q13.3 has been linked to early hypotonia, cognitive, and language impairments, autism-like behaviors, lymphedema, and dysmorphic traits. Several studies indicated that seizures were more prevalent if a de novo deletion occurred on the maternal rather than paternally inherited chromosome 22 [119]. Shank3 knockout mice exhibit autistic traits, as well as anomalies in the corticostriatal network. Patients with ASD have been shown to have SHANK1 deletions [120] and SHANK2 mutations [121].

## 5. Treatment Approaches

Antiseizure medications ought to rescue cognitive flexibilities and comorbidities in ASD with epilepsy models induced by E/I imbalance. This approach certainly is successful in animal models. For example, a mouse model for common DRS was treated by clonazepam [122], attenuating cognitive actions [67], but this treatment does not work in humans. In a randomized controlled experiment, it was discovered that exposure to cannabidiol (CBD) and its metabolites increased dosage proportionality in individuals with DRS. Clobazam (CLB) with three dosages of CBD (5, 10, and 20 mg/kg/d) resulted in a PK interaction, increasing plasma levels of N-desmethylclobazam (N-CLB) in patients. It is possible that elevated levels of N-CLB might contribute to both benefit (antiseizure) and unfavorable effects (sedation, exhaustion) associated with CBD treatment [123]. The side effects of CBD were more severe than those of a placebo. However, it was typically well tolerated at doses of 5–20 mg/kg/d, and the safety profile observed in open-label studies was consistent with the safety results. All three CBD dosages were typically well tolerated [124]. In recent study, CBD and *Cannabis sativa* extracts were examined using a 6 Hz corneal stimulation mice model [125]. This research offers the comprehensive qualitative and quantitative characterization of terpenes in extracts, with a special focus on K2 hemp oil, which was prepared while conserving volatile components. According to the findings, terpenic components have a role in increasing the antiepileptic action of cannabinoids in K2 hemp oil, compared to K1 and pure CBD, even if small chemicals in extracts might contribute to overall activity. The findings imply that both cannabinoids and terpenes found in oil extracts should be evaluated as potential therapeutic agents for epileptic seizures and epilepsy [126]. Conventional anticonvulsant drugs are ineffective in treating cognitive disorders characterized by an imbalance of E/I [127]. This suggests that the process of cognitive impairment is likely to be more complex than modification of the E/I equilibrium of ASD and epilepsy. The E/I equilibrium theory should be reviewed in terms of existing hypotheses to categorize fundamental possible pathways.

The use of antiseizure medication therapy as a treatment for ASD symptoms has not been well studied in controlled studies. Several clinical studies have shown that valproic acid may be used to treat autistic children who have clinical seizures or epileptiform EEG identified abnormalities [128]. Patients with abnormal EEG or seizure history were classified as responders in an open study with valproic acid in which 10 of 14 individuals displayed improvement in key symptoms of autism and accompanying emotional instability, hyperactivity, and aggressiveness [129]. Levetiracetam has been shown to aid autistic individuals with hyperactivity, irritability, emotional inconsistency, and aggressiveness [130]. Almost 7% of the patients quit taking levetiracetam due to abnormal behavior, and this was the most prevalent reason for discontinuation. These behavioral changes included suicide thoughts and aggressiveness toward others. Levetiracetam may aggravate behavioral symptoms in individuals with a genetic susceptibility to psychiatric condition. The individuals with generalized epilepsy were more likely to stop using levetiracetam due to behavioral disorders than those with localized epilepsy [131]. However, the “rebound effect” occurs when people abruptly stop using antiseizure medications and experience an increase in the frequency and severity of epileptic episodes. Delta band power in the postictal component of a seizure was linked to the incidence of seizures following levetiracetam discontinuation. Allopregnanolone levels in the hippocampus were shown to be positively associated with seizure and delta band power. Recent findings show that the seizure postictal component has a role in the rebound effect, which is characterized by an imbalance in hippocampus neurosteroid levels [132].

A ketogenic diet (KD) is a high-fat, moderate-protein, low-carbohydrate dietary intervention therapy in neurological disorders such as epilepsy and ASD [133]. The neuroprotective effect of a KD in ASD, which is probably achieved by improved energy metabolism, decreased levels of oxidative stress, regulation of neurotransmitters, suppression of the mammalian target of rapamycin (mTOR) signaling pathway, and regulation of the gut microbiota, implies that KD is most likely a safe and effective therapy for ASD [134].

Nevertheless, negative consequences such as growth retardation limit the usage of KD. Previously, it was found that children maintained on KD for three months have decreased ghrelin levels [135]. Recent study discovered a sustained decrease in ghrelin levels in children with refractory epilepsy who were treated with KD and received a sufficient calorie intake. The reduced amount of ghrelin may be associated to the slower development of children on KDs [136].

Health care quality and disease management are improved by the use of e-health technologies. In a previous study, researchers developed a KD management app and a website to provide information to caregivers about this dietary therapy for children with refractory epilepsy. From January to March 2016, a questionnaire survey was performed by 40 different families. Findings suggest that use of e-health apps in the daily management of the ketogenic diet might be a useful strategy, especially during the continuing pandemic crisis of COVID-19 [137]. In the absence of clinical studies, no definitive recommendations regarding any of these therapies can be made. Among individuals with ID, ASD, and dysmorphic characteristics, CMA (chromosomal microarray analysis) has shown the greatest diagnostic yield (66.7%), suggesting its value as a first level diagnostic genetic test in this population [138]. As a result of these advances, many novel ASD causing de novo mutations have been found [139].

## 6. Conclusion

There is a substantial gap in understanding of the relationship between epilepsy and ASD and research that addresses this issue is limited. Presently, it is unclear whether epileptiform interictal discharges and ASD are epiphenomena of the physiological process or whether their connection results from causation. In this review, the relationship between ASD, epilepsy, and ID was studied along with whether they shared similar neurodevelopment pathways. There are numerous characteristics of ASD and epilepsy, both hereditary and acquired, which may be categorized as originating from atypical connectivity. It has been discussed that several proteins involved in the synaptic plasticity of autism and epilepsy and that their genes are disrupted as a result of genetic mutations. It is important to determine whether these two phenomena are associated: if they are linked, new pharmacological therapies are required for the protection patient cognition and behavior. Alternatively, if unlinked, patients should not be treated with anticonvulsants due to their low-risk benefit ratio. This concept may lead to a new avenue for a better understanding of the known association between autism and epilepsy including, but are not limited to, gene transcriptional regulation, cellular growth, and synapse development, stability, and function. Regardless of the complicated relationship between these two conditions, it is suggested that an underlying pathophysiological pathway is common to epilepsy and autism.

## Figures and Tables

**Table 1:** Correlating genetic disorder in both epilepsy and ASD.

Genetic syndromes	Coexistence (*p* value)	Proportion of epilepsy % (*n*)	*p* value	Proportion of ASD % (*n*)	*p* value
Tuberous sclerosis complex		10.8% (*n* = 103) [73] 70-80% (*n* = 138) [74]	(*p* < 0.05) (*p* < 0.05)	25-50% (*n* = 32) [75]	(*p* < 0.05)
Down syndrome	(*n* = 40), (*p* < 0.001)	8% (*n* = 146) 1%–13% (*n* = 104) [73]	(*p* < 0.05) (*p* < 0.05)	5.8% [76] 3.1% (*n* = 36) [72] 18.2% (*n* = 123) [77]	(*p* < 0.05) (*p* < 0.01)
Dravet syndrome		100%(*n* = 18) 100% (*n* = 20) [78]	(*p* < 0.01) (*p* < 0.05)	61.5% (*n* = 18) 24.3% (*n* = 37) [79]	(*p* < 0.01) (*p* < 0.01)
Fragile X syndrome	28.1% (*n* = 57) (*p* < 0.05)	11.8%–18% (*n* = 41) [80]	(*p* < 0.001)	30% [81] 21% (*n* = 75)	(*p* < 0.001)
Rett syndrome		61% (*n* = 313) [82]	(*p* < 0.05)	Transitory autism features (*n* = 12) [83]	(*p* < 0.05)
Pitt–Hopkins syndrome		50%(*n* = 26) [84]	(*p* < 0.05)	100% (*n* = 12) [85]	(*p* < 0.05)
Hypomelanosis of Ito syndrome	(*n* = 41) (*p* < 0.01) [86]	37%–53% [87] 11.5%–50%(*n* = 41) 49% (*n* = 4) [88]	(*p* < 0.01) (*p* < 0.05)	64% (*n* = 76) [89] 10% of patients with ID (*n* = 4) [90]	(*p* < 0.05) (*p* < 0.05)
Smith–Lemli–Opitz syndrome	(*n* = 85) (*p* < 0.01)	(*n* = 85) [91]	(*p* < 0.01)	53% (*n* = 88) [92]	(*p* < 0.05)
Sotos syndrome		Rare (*n* = 61) [93]	(*p* < 0.05)	41% (*n* = 7) [94]	(*p* < 0.05)
Angelman syndrome	(*n* = 4) (p <0.05) [95]	100% (*n* = 19) [96] 85% (*n* = 18) [97]	(*p* < 0.01) (*p* < 0.05)	42% (*n* = 19) [98] 1.9% (*n* = 12) [99]	(*p* < 0.05) (*p* < 0.001)

## Data Availability

All the study data is available on request.
